# Laser Speckle Flowmetry for the Prognostic Estimation Study of Permanent Focal Ischemia in Mice

**DOI:** 10.1155/2022/1729255

**Published:** 2022-09-20

**Authors:** Cong Li, Guoqing Zhou, Ming Yang, Qin Tang, Liangzhen Zhu, Yuan Wang

**Affiliations:** ^1^School of Medical Information Engineering, Shandong First Medical University & Shandong Academy of Medical Sciences, Taian, 271000 Shandong, China; ^2^Department of Neurology, Second Affiliated Hospital, Shandong First Medical University & Shandong Academy of Medical Sciences, Taian, 271000 Shandong, China; ^3^Department of Ultrasound, Shandong Provincial Hospital, Affiliated to Shandong First Medical University, Jinan, 250021 Shandong, China; ^4^Department of Neurology, Shandong Provincial Hospital, Affiliated to Shandong First Medical University, Jinan, 250021 Shandong, China

## Abstract

The distal middle cerebral artery occlusion (dMCAO) model that mainly targets the cortex and causes low mortality is developed for the study of permanent focal ischemia, and it is highly appropriate for the study in the aged population. The two most common methods used to establish dMCAO models are dMCAO alone and dMCAO plus ipsilateral common carotid artery occlusion (CCAO). Up to now, studies on the prognosis of the two types of dMCAO models and the accuracy of cerebral blood flow (CBF) in predicting prognosis have not yet been reported. In the present study, we established permanent focal ischemia models in two groups of aged mice by dMCAO alone or by dMCAO plus ipsilateral common carotid artery occlusion (CCAO). CBF was evaluated by laser speckle flowmetry (LSF) before and after surgery. Cerebral infarction was assessed by TTC staining at day 2 after surgery and MAP2 staining at day 21 after surgery. In addition, behavioral outcomes were evaluated using the modified Garcia scoring system, adhesive removal test, and foot-fault test. Our results showed that compared with those in the dMCAO alone group, the mice in the dMCAO plus CCAO group had a larger cerebral infarct size and more severe neurological deficits. According to the results of the correlation analysis, the area of the ischemic core region on CBF imaging in the dMCAO group was helpful in predicting the infarct volume. In addition, the total CBF of the ischemic area in the dMCAO plus CCAO group showed a significant correlation with Garcia scores 3 days after surgery, but there was no significant correlation of CBF imaging with the foot-fault test 7 days after surgery. These results suggest that the total CBF of the ischemic area might be helpful to predict the severity of neurological damage at the acute stage.

## 1. Introduction

Stroke is the second leading cause of both disability and death worldwide and poses a staggering burden at both societal and individual levels [1, 2]. Of all strokes, approximately 87% are ischemic strokes, and the global incidence of ischemic stroke is approximately 101.3 per 100,000 population [2]. A variety of animal models, particularly in rodents, have been developed to investigate the underlying pathophysiological mechanisms and explore therapeutics for ischemic stroke [3, 4]. Different murine models of ischemic stroke lead to different degrees of cerebral injury and neurological deficits [5, 6]. The distal middle cerebral artery occlusion (dMCAO) model is a rodent stroke model that mainly targets the cortex and exhibits low mortality, so it is highly appropriate for the studies in the aged population [7]. Permanent distal MCA occlusion alone or plus ipsilateral common carotid artery occlusion (CCAO) are two common methods for permanent focal ischemia model preparation. Nevertheless, no studies have examined the distinctions of the two types between operations on cerebral infarct size and neurological deficits.

Laser speckle flowmetry (LSF) provides a rapid and wide-field characterization of light scattering particle motion through camera exposure, and it has become widely used as a blood flow imaging tool for measuring blood flow in the brain, skin, retina, mesenterium, and kidney [8–10]. Cerebral blood flow (CBF), measured by LSF, is widely used in studies of experimental stroke models [11, 12]. In a cerebral ischemia model, cerebral artery occlusion significantly decreases local cerebral blood flow, leading to neuronal death and neurological impairments [13–15]. Therefore, CBF detection is commonly applied to assess the blockage of the middle cerebral artery during the cerebral ischemia model-making process [14, 16]. In addition, low CBF was considered to be correlated with poor behavioral or cognitive functions after stroke [17]. However, studies on the correlations between the CBF of dMCAO mice and prognosis have not yet been reported. Thus, whether the LSF imaging of CBF is useful for the prognostic estimation of the dMCAO model remains to be investigated.

In this study, we compared and contrasted the CBF and cerebral injury of the two dMCAO models. The results indicate that CBF, cerebral infarct, and neurological functions vary widely between the dMCAO alone group and the dMCAO plus CCAO (dMCAO+CCAO) group. In addition, the CBF-related parameters from LSF imaging did not have a strong correlation with the severity of cerebral infarction and functional impairments in the dMCAO models.

## 2. Materials and Methods

### 2.1. Animals and Experimental Design

Adult male C57BL/6J (9 months old) mice were used to model permanent focal ischemia. All experimental procedures were performed according to the Guide for the Care and Use of Laboratory of Shandong First Medical University. With ad libitum access to food and water, all animals were housed in a 12-hour light/dark cycle with controlled temperature and humidity. In all experiments, investigators were blind to the assignment of the experimental groups.

### 2.2. Murine Model of Distal Focal Ischemia

According to previous researches, distal permanent focal cerebral ischemia was produced by permanent distal MCA occlusion (dMCAO) alone and dMCAO plus ipsilateral common carotid artery (CCA) occlusion [18, 19]. In brief, with a rodent ventilator (RWD, China), mice were anesthetized with 3% isoflurane and maintained anesthesia with 1.5% isoflurane in a mixture of 30% O_2_/70% N_2_O. First, an incision was made in the midline neck skin, and then, the left CCA was isolated and occluded by ligation. Then, the temporal muscle was coagulated with electrical current through an incision between the left eye and the ear. A burr hole was opened, and a craniotomy was performed to expose the distal part of the MCA. The distal MCA was occluded by microbipolar coagulation after cutting open the dura mater. Finally, the skin was sutured. Throughout the surgery, the mice's rectal temperature was monitored and kept at 37 ± 0.5°C. A sham operation involved the same anesthesia and procedure but did not involve CCAO or dMCAO. The mice in the dMCAO alone group received the same anesthesia and dMCAO procedures but without CCAO.

### 2.3. Laser Speckle Contrast Imaging of Cerebral Blood Flow (CBF)

The CBF of all mice was measured both before and after surgery. Real-time two-dimensional CBF was measured using laser speckle flowmetry (RFLSI III, RWD, CN), as previously reported [18]. The mouse was anesthetized and placed in the prone position. Then, the skull was exposed by an incision of the skin along the midline of the scalp. The LSF was elevated to an appropriate height above the skull surface. A whole-brain scan was performed by using LSF. To evaluate CBF changes, regions of interest (ROIs) were selected manually, and data were analyzed using LSCI_V 1.0.0 software (RWD, CN).

**Figure 1:**
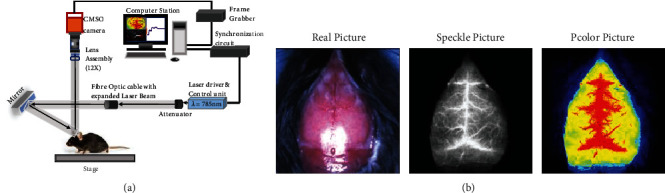
Schematic diagram of the laser speckle imaging system. (a) Schematic diagram of the RFLSI III laser speckle perfusion imager. Speckle images are acquired by the laser at 785 nm. Scattered laser light from the brain is captured by a CMSO camera, followed by digitization by a computer-based frame grabber. (b) Representative images of cerebral blood flow. Left image: real picture of the mouse brain. Middle image: laser speckle contrast imaging of cerebral blood flow. Right image: pseudocolor image of the middle image. Each image covers an area of 1.2 × 1.2 cm^2^, corresponding to 2048 × 2048 pixels.

Figure 1(a) presents the optical configuration of the laser speckle flowmetry used in this study. Briefly, high-resolution blood flow speckle images were recorded by a CMSO camera while the skull is illuminated by a laser at a wavelength of 785 nm. As shown in Figure 1(b), three different types of images were acquired with the laser speckle imaging system: real pictures, speckle pictures, and pcolor pictures. The regions where the CBF exhibits less than 30% of baseline CBF were defined as ischemic core areas. Meanwhile, the regions where the CBF exhibits 30–50% of baseline CBF were defined as penumbra [19]. LSCI_V 1.0.0 software (RWD, CN) was used to determine the regions and calculate the areas.

### 2.4. Measurements of Infarct Volume or Tissue Loss

2,3,5-Triphenyltetrazolium chloride (TTC) staining was used to assess the infarct volume 2 days after surgery, and microtubule-associated protein 2 (MAP2) immunofluorescence staining was used to evaluate the tissue loss 21 days after surgery. According to our previous literature [20], for TTC staining, the mouse forebrains were removed at 48 hours after surgery and sliced into 1-mm-thick brain slices, followed by immersion in 2% TTC/saline solution at 37°C for 10 minutes. The normal brain area was stained red, while the infarct area was not stained. As a neuron-specific marker, MAP2 was used to label surviving neurons. In brief, the forebrains of mice were removed at 21 days after surgery, and a routine procedure was performed for frozen tissue sections (25-*μ*m-thick). Brain slices from 8 classical layers were extracted to perform routine immunofluorescence staining with anti-MAP2 antibody (Santa Cruz Biotechnology). To be specific, selected brain sections were incubated with MAP2 primary antibody (1 : 200, Abcam, USA) overnight at 4°C and then with suitable secondary antibodies (488-conjugated donkey antirabbit IgG, 1:800, Jackson Immunoresearch, USA). A confocal microscope (Nikon, Japan) was used to capture the pictures. Infarct volume or tissue loss was quantified from the stained sections by using ImageJ software. The infarct volume was calculated by subtracting the noninfarcted volume of the ipsilateral hemisphere from the volume of the contralateral hemisphere.

### 2.5. Behavioral Tests

A blinded behavioral test was performed on mice to evaluate their sensorimotor functions. In this study, we assessed comprehensive neurological function following dMCAO using the Garcia scoring system [18]. The modified Garcia scoring system consists of five kinds of items (3 points/item, maximal score = 15), including body proprioception, forelimb walking, limb symmetry, lateral turning, and climbing.

In the adhesive removal test [21], adhesive tapes of the same size (0.3 × 0.4 cm^2^) are applied with equal pressure to the animal paws. The seconds to contact and remove each adhesive tape will be recorded, with a maximum of 60 s. Mice will be trained once daily before surgery for 3 days and regularly tested (3 trials per day per mouse with 15-minute interval between different mice) after surgery at the indicated time points. The mean latency of three trials to remove the tapes will be calculated.

The foot-fault test [16, 22] focuses on deficits in motor control. Mice will be placed on an elevated steel grid with an opening of 2.25 cm^2^ (1.5 × 1.5 cm^2^) and videotaped for 1 minute. Mice will be pretrained for 3 days before surgery and regularly tested after surgery at the indicated time points. The number of total steps and foot faults of the right limbs will be counted by a blinded investigator. Errors versus total steps taken by the contralateral limbs were used to calculate foot-fault percentages.

### 2.6. Statistical Analysis

All data are presented as means ± SEM. The differences among means of groups were analyzed using two-way ANOVA or Student's two-tailed *t* test with the normally distributed data, and the correlation analyses were performed using Pearson *correlation* in GraphPad Prism software 7.0. A *p* value < 0.05 was considered statistically significant.

## 3. Results

### 3.1. Cerebral Blood Flow Shows a Significant Difference between the dMCAO Alone Group and the dMCAO+CCAO Group

**Figure 2:**
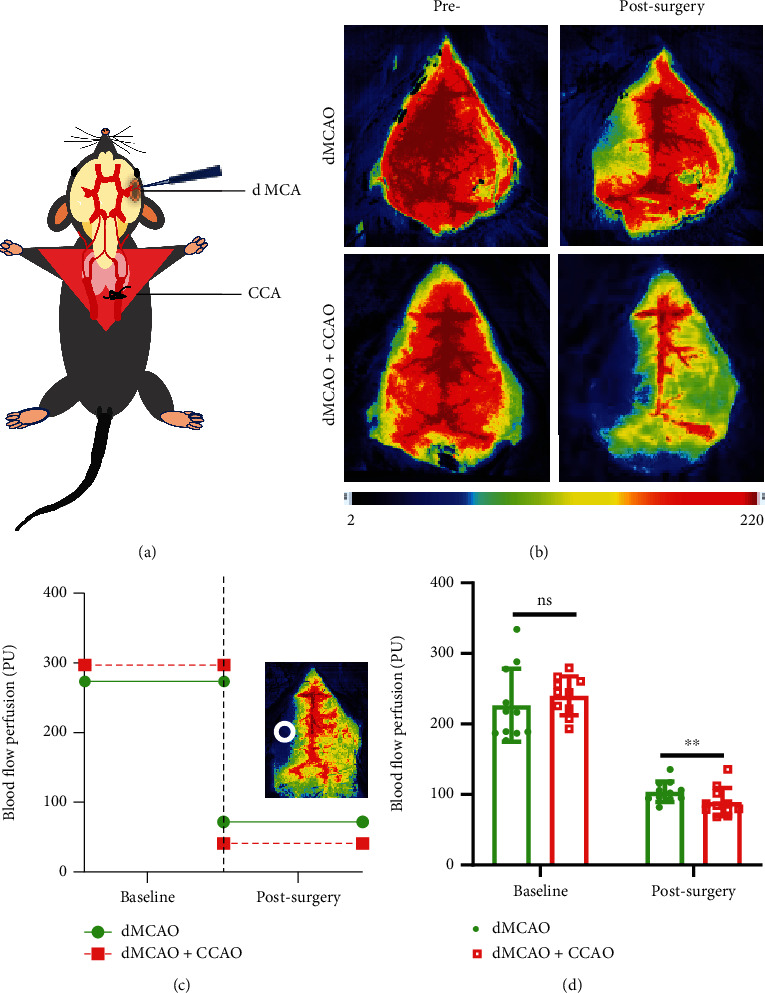
Laser speckle contrast imaging of different operations for distal MCAO. Cerebral blood flow (CBF)-related data were collected by laser speckle flowmetry 5 minutes after the operation. (a) Schematic diagram of the surgery. (b) Representative laser speckle images of cerebral blood flow (CBF) recorded from dMCAO and dMCAO+CCAO mice brain. (c) Representative line graph of CBF in the infarct core area before and after surgery. (d) Quantification of CBF in the infarct core area before and after surgery. *n* = 10–11. Data are expressed as the mean ± SEM. The differences between groups were analyzed using the Student's two-tailed *t* test with the normally distributed data. ns: no significance, ∗∗*p* ≤ 0.01, ∗∗∗*p* ≤ 0.001.

CBF changes could be monitored by using LSF. First, we wished to explore the CBF differences between the dMCAO alone group and the dMCAO+CCAO group. As shown in Figure 2(a), distal focal cerebral ischemia was produced by permanent distal MCA occlusion (dMCAO) alone and dMCAO plus ipsilateral CCA occlusion (dMCAO+CCAO). CBF was detected before and 5 minutes after surgery [23]. The whole brain showed a high blood flow perfusion pattern before surgery, whereas the CBF on the left side decreased dramatically after surgery in both groups (Figures 2(b) and 2(c)). There were no visible differences in the baseline CBF of the left-side brain between the dMCAO group and the dMCAO+CCAO group. However, the blood flow perfusion in the left MCA territories decreased sharply after surgery, and the CBF in the left-side brain of the mice in dMCAO+CCAO group mice was significantly lower than that in dMCAO alone group (Figure 2(d), 103.99 ± 14.58 PU in dMCAO group and 84.6 ± 13.67 PU in the dMCAO+CCAO group, *p* < 0.01). These results confirmed that the CBF in the left side of the brain was significantly different between the dMCAO+CCAO mice and the dMCAO mice.

### 3.2. The dMCAO+CCAO Operation Results in Enlarged Brain Infarcts Compared to the dMCAO Operation

**Figure 3:**
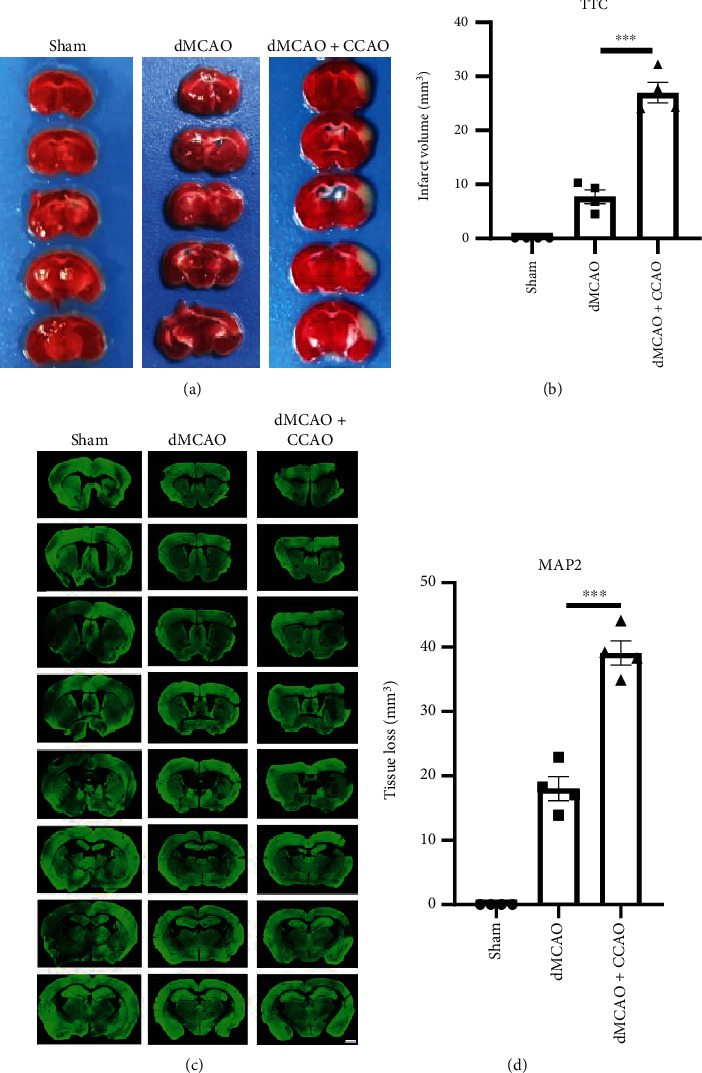
Infarct volume and tissue loss caused by two kinds of operations in the distal MCAO model. (a) Representative images of 2,3,5-triphenyltetrazolium chloride (TTC) staining for infarct measurement 2 days after surgery. (b) Quantification of infarct volume by TTC staining. *n* = 4. (c) Brain sections were immunostained by MAP2 21 days after surgery. Representative brain slices with MAP2 showed reduced cerebral tissue atrophy at 21 d after ischemia. (d) Quantification of tissue loss by MAP2 immunostaining. *n* = 4. Data are expressed as the mean ± SEM. The differences between groups were analyzed using the Student's two-tailed *t* test with the normally distributed data. ∗∗∗*p* ≤ 0.001.

To explore the effects of different surgical approaches on brain infarcts, we used TTC staining 2 days after surgery for infarct volume calculation and MAP2 immunofluorescence staining 21 days after surgery for tissue loss assessment. No infarct area was observed in the sham group while noticeable infarct areas were observed in both model groups, as evidenced by TTC negatively stained areas (Figure 3(a)). In addition, compared to dMCAO mice, dMCAO+CCAO mice exhibited significantly larger infarct volumes (Figure 3(b). dMCAO mice 7.74 ± 2.59 mm^3^ vs. dMCAO+CCAO mice 27 ± 3.82 mm^3^, *p* < 0.001). Similarly, brain tissue loss was observed in MAP2-stained sections of the two model groups (Figure 3(c)), which was significantly larger in the dMCAO+CCAO group than in the dMCAO group (Figure 3(d). MCAO mice 18.02 ± 3.73 mm^3^ vs. dMCAO+CCAO mice 39.08 ± 3.8 mm^3^, *p* < 0.001). Taken together, these data indicate that the dMCAO+CCAO operation results in enlarged brain infarcts compared to the dMCAO operation.

### 3.3. Tissue Loss May Be Correlated with the Area of Ischemic Core Regions on the CBF Imaging in the dMCAO Group

**Figure 4:**
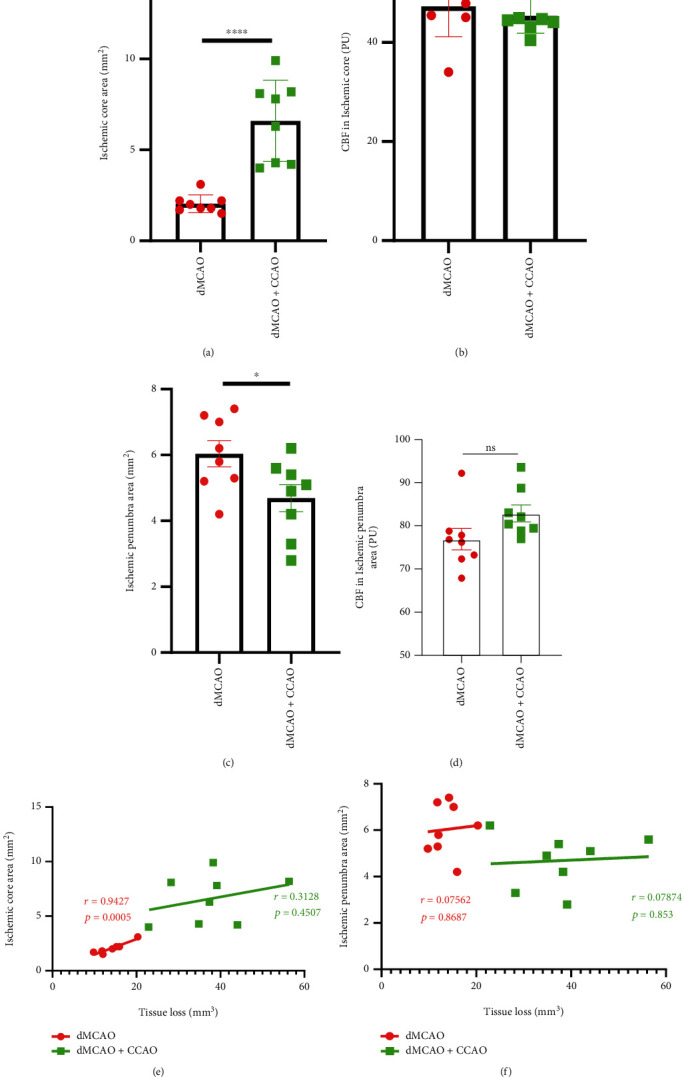
The correlation between tissue loss and CBF in the ischemic core or penumbra area. Tissue loss was measured by MAP2 immunostaining 21 days after surgery. CBF-related data were collected by laser speckle images 5 minutes after the operation. Quantification of areas of ischemic core region (a) and penumbra region (c) by laser speckle images. Comparison of the overall mean CBF in the ischemic core area (b) and penumbra area (d) between the dMCAO and dMCAO+CCAO groups. The correlation between the volume of tissue loss and the area of ischemic core regions (e) or penumbra regions (f). *n* = 8. Data are expressed as the mean ± SEM. The differences between groups were analyzed using the Student's two-tailed *t* test with the normally distributed data. The correlation analyses were performed using *Pearson correlation.* ns: no significance, ∗*p* ≤ 0.05, ∗∗∗*p* ≤ 0.001.

To investigate whether laser speckle imaging of CBF could help predict the severity of ischemic injury, we evaluated the correlation between the ischemia-related area on CBF imaging and the volume of tissue loss 21 days after surgery. The laser speckle imaging of CBF indicated that compared with dMCAO alone ones, dMCAO+CCAO mice had a significantly larger area of the ischemic core region (Figure 4(a), 2.04 ± 0.49 mm^2^ in dMCAO alone group and 6.6 ± 2.24 mm^2^ in dMCAO+CCAO group, *p* < 0.0001) but a smaller area of ischemic penumbra (Figure 4(c), 6.04 ± 1.12 mm^2^ in dMCAO alone group and 4.69 ± 1.17 mm^2^ in dMCAO+CCAO group, *p* < 0.05). There were no significant differences in the CBF of the ischemic core area and penumbra area between the dMCAO group and the dMCAO+CCAO group (Figures 4(b) and 4(d)). The volume of tissue loss 21 days after surgery exhibited a positive correlation with the ischemic core area of CBF imaging in dMCAO mice (Figure 4(e), *r* = 0.9427, *p* = 0.0005). Nevertheless, this correlation was not remarkable in dMCAO+CCAO mice. The correlation between tissue loss and the ischemic penumbra area was also not remarkable in either group (Figure 4(f)). These data suggest that the area of ischemic core region on CBF imaging is helpful in predicting the infarct volume after dMCAO surgery.

### 3.4. dMCAO+CCAO Mice Exhibit Greater Deterioration in Sensorimotor Functions than dMCAO Mice

**Figure 5:**
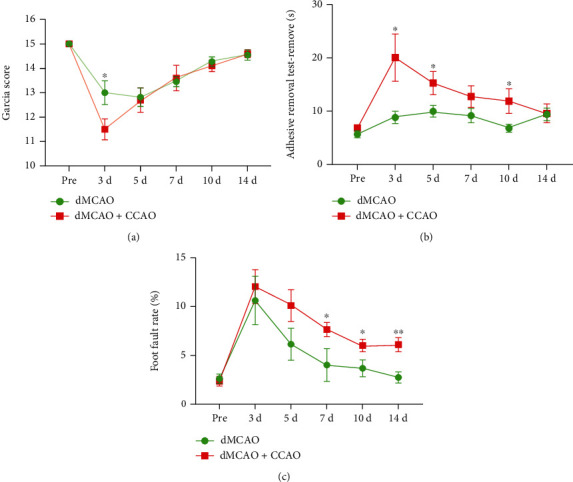
Neurobehavioral functions were different between the dMCAO and dMCAO+CCAO groups. Three neurobehavioral tests were performed to assess poststroke neurological deficits up to 14 d after dMCAO or dMCAO+CCAO surgery. (a) Neurological deficiencies were evaluated based on the criteria of Garcia assessments. The dMCAO+CCAO group mice performed differently on the Garcia score than the dMCAO group mice on postoperative day 3. (b) The adhesive removal test and foot-fault test (c) were performed to assess sensorimotor impairment after surgery. *n* = 10–11. Data are expressed as the mean ± SEM. The differences among groups were analyzed using the two-way ANOVA test with the normally distributed data. ∗*p* ≤ 0.05, ∗∗*p* ≤ 0.01.

To assess the neurofunctional differences between the dMCAO alone group and the dMCAO+CCAO group, we employed the Garcia scoring system, the adhesive removal test, and the foot-fault test to examine the neurobehavioral capacity before and up to 14 days after surgery. Compared with dMCAO mice, dMCAO+CCAO mice showed lower scores in comprehensive neurological impairment at day 3 after surgery (Figure 5(a), *p* < 0.05). In the adhesive removal test, dMCAO+CCAO mice exhibited prolonged sensorimotor deficits lasting for 14 days after surgery (Figure 5(b), *p* < 0.05). Similarly, the dMCAO+CCAO mice exhibited a higher foot-fault rate than the dMCAO mice, which lasted for at least 14 days after surgery (Figure 5(c), *p* < 0.05). These results demonstrate that the dMCAO+CCAO operation results in more severe neurological deficits in mice than the dMCAO operation.

### 3.5. Decreased CBF in the Sensorimotor Cortex May Be Correlated with Neurological Impairments in the dMCAO+CCAO Group

**Figure 6:**
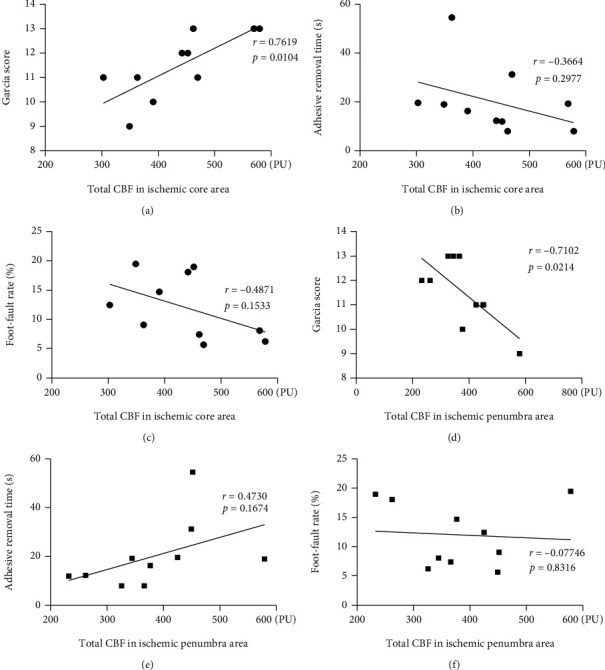
The correlation between neurobehavioral functions and total CBF in the ischemic core or penumbra area in the dMCAO+CCAO group. Functional neurological evaluation was performed 3 days after dMCAO+CCAO surgery. The correlation between the total CBF in the ischemic core area and Garcia score (a), adhesive removal time (b), or foot-fault rate (c). The correlation between the total CBF in the ischemic penumbra area and Garcia score (d), adhesive removal time (e), or foot-fault rate (f). The correlation analyses were performed using *Pearson correlation.*

Next, we assessed whether the neurological impairments after dMCAO+CCAO surgery were linked to CBF-related indicators by laser speckle imaging. CBF imaging was captured 5 minutes after surgery, and the most representative time points were selected for neurological function tests (day 3 for the Garcia scoring and the adhesive removal test, day 7 for the foot-fault test). Pearson correlation was used for the correlation detection. As shown in Figures 6(a) and 6(d), the Garcia scores exhibited a positive correlation with the total CBF in the ischemic core area (*r* = 0.7619, *p* = 0.0104) and an inverse correlation with the total CBF in the ischemic penumbra area (*r* = 0.7102, *p* = 0.0214). There were no significant correlations between total CBF in the ischemic core (or penumbra) area and adhesive removal time in the adhesive removal test (Figures 6(b) and 6(d)) or foot-fault rate in the foot-fault test (Figures 6(c) and 6(f)). In addition, the areas of ischemic core (or penumbra) regions also exhibited no significant correlation with the neurological functions (data was not shown). These results suggest that the total CBF of the ischemic region may be to some extent correlated with neurological impairments in the acute phase of ischemic injury, but CBF imaging cannot accurately indicate the severity of long-term neurological damage.

## 4. Discussion

The goals of the present study were to compare the outcomes of two types of operations for permanent focal cerebral ischemia in mice and to evaluate the possibility of prognostic estimation through laser speckle flowmetry imaging for cerebral ischemia. In this study, we established a permanent focal cerebral ischemia model by two methods: dMCAO alone and dMCAO plus CCAO. Compared with mice in the dMCAO alone group, mice in the dMCAO+CCAO group had a larger cerebral infarct size and more severe neurological deficits. According to the results of the correlation analysis, the area of the ischemic core region on CBF imaging in the dMCAO group had a significant correlation with the infarct volume. Thus, the severity of ischemia injury after dMCAO surgery could be largely predicated through CBF imaging by the operator. In addition, the total CBF of the ischemic region in the dMCAO+CCAO group showed a significant correlation with Garcia scores 3 days after surgery, and there was no significant correlation of CBF imaging with the results of foot-fault test 7 days after surgery. To sum up, the CBF imaging cannot accurately indicate the infarct volume and the severity of long-term neurological damage after dMCAO+CCAO surgery in mice. The total CBF of the ischemic area could help the operator to predict the severity of acute neurological damage.

In this study, we chose the dMCAO mouse model for experimental permanent focal ischemia. Compared with other cerebral ischemia models such as the routine intraluminal thread MCAO model, distal MCAO has high survivability and reproducibility of infarct lesions [24]. This method is suitable for the observation of ischemic injury involving neuronal death and neurological functional impairments. In addition, the distal MCAO model can be performed without regard to the diameter of the middle cerebral artery, which could influence the stability of the model [24]. The distal MCAO model could be established by distal MCA occlusion alone or dMCAO plus CCA occlusion. Previous studies reported that the infarct regions of dMCAO without CCAO mice were restricted to the cortex [25]. The small range of the infarct area may limit the application of the dMCAO alone model. The present study is the first to evaluate the differences between the two types of operations for the distal MCAO model. From the results of TTC staining 2 days after surgery and MAP2 staining 21 days after surgery, noticeable infarct areas and tissue loss were observed in both model groups, and the dMCAO+CCAO mice presented a larger infarct volume than dMCAO alone mice. The larger infarct area in dMCAO+CCAO mice might be related to the impairment of the collateral circulation by ipsilateral CCA occlusion [24, 25].

The Garcia score, adhesive removal tests [21], and foot-fault tests [23] were used to assess the neurobehavioral capacity before and up to 14 days after surgery. These behavior tests are classical methods for sensorimotor deficit assessment. Consistent with the infarct volume results, mice in the dMCAO+CCAO group presented more severe neurological deficits throughout 14 days after surgery than those in the dMCAO alone group. Therefore, we can conclude that the two types of operations of the dMCAO model result in varying degrees of cerebral injuries and neurobehavioral deficits, which could meet the researchers' different demands for the permanent focal ischemia model.

With the advantage of full-view field imaging of surface blood flow, LSF has been widely used to identify ischemic tissue and to predict infarct volume in rodent cerebral ischemia models [26, 27]. This study is the first to use LSF to monitor CBF changes in two types of operations for a distal MCAO model of peri-ischemia. The ischemic core and penumbra regions are defined as the regions where CBF decreases to less than 30% and 30–50% of baseline, respectively [28]. Several important novel findings were made according to the CBF data. First, compared to the dMCAO alone group, the ischemic core area was significantly larger, while the CBF in the whole left hemisphere was lower in the dMCAO+CCAO group after surgery. This might be due to the lower arterial blood pressure by additional occlusion of the CCA during ischemia [29]. Second, tissue loss might be correlated with the size of the ischemic core area on the CBF imaging in the dMCAO alone group, but such association was not evident in the dMCAO+CCAO group. Third, decreased CBF in the sensorimotor cortex may be correlated with neurological impairments in the dMCAO+CCAO group. The infarction of the sensorimotor cortex leads to neurological impairments, and MCA is the main artery that supplies blood to the sensorimotor cortex [30]. In this study, we used Garcia scoring scale to evaluate comprehensive neurological functions following ischemic injury, and the Garcia score exhibited a positive correlation with the total CBF in the ischemic core area and an inverse correlation with the total CBF in the ischemic penumbra area. These results suggest that the total CBF in the ischemic area may help predict the early lesion of the sensorimotor cortex.

In conclusion, the dMCAO+CCAO mice exhibited marked differences in CBF-related parameters compared to those in the dMCAO alone mice, and a strong correlation was also found between CBF and the cerebral infarction and the functional outcomes. In addition, the CBF imaging can help to predict the infarct volume in the dMCAO alone mice and the severity of neurological damage at the acute stage in the dMCAO+CCAO mice. These findings may help the operators to predict the prognosis of the permanent focal ischemia to a certain degree.

## Data Availability

The original datasets generated for this study are available on request to the corresponding author.
